# A Review of Recent Advances in Microbial Fuel Cells: Preparation, Operation, and Application

**DOI:** 10.3390/biotech11040044

**Published:** 2022-09-30

**Authors:** Jianfei Wang, Kexin Ren, Yan Zhu, Jiaqi Huang, Shijie Liu

**Affiliations:** 1 Department of Chemical Engineering, SUNY College of Environmental Science and Forestry, Syracuse, NY 13210, USA; 2 The Center for Biotechnology & Interdisciplinary Studies (CBIS), Rensselaer Polytechnic Institute, Troy, NY 12180, USA

**Keywords:** microbial fuel cells, wastewater treatment, lignocellulosic biomass, genetic engineering, electrode modification, value-added products

## Abstract

The microbial fuel cell has been considered a promising alternative to traditional fossil energy. It has great potential in energy production, waste management, and biomass valorization. However, it has several technical issues, such as low power generation efficiency and operational stability. These issues limit the scale-up and commercialization of MFC systems. This review presents the latest progress in microbial community selection and genetic engineering techniques for enhancing microbial electricity production. The summary of substrate selection covers defined substrates and some inexpensive complex substrates, such as wastewater and lignocellulosic biomass materials. In addition, it also includes electrode modification, electron transfer mediator selection, and optimization of operating conditions. The applications of MFC systems introduced in this review involve wastewater treatment, production of value-added products, and biosensors. This review focuses on the crucial process of microbial fuel cells from preparation to application and provides an outlook for their future development.

## 1. Introduction

With population growth and industry development, the global energy demand is increasing rapidly. At present, human lives and industrial productions mainly depend on fossil fuels. However, gaseous emissions from the combustion of fossil fuels lead to air pollution and the greenhouse effect. In addition, the massive consumption of fossil energy will also result in a potential energy crisis [1]. Although clean energy sources, such as wind and nuclear energy, have been widely developed and deployed, no solution can replace fossil fuels independently [1,2]. Therefore, it is still necessary to further develop renewable energy alternatives to achieve efficient environmental protection and sustainable economic development.

**Figure 1:**
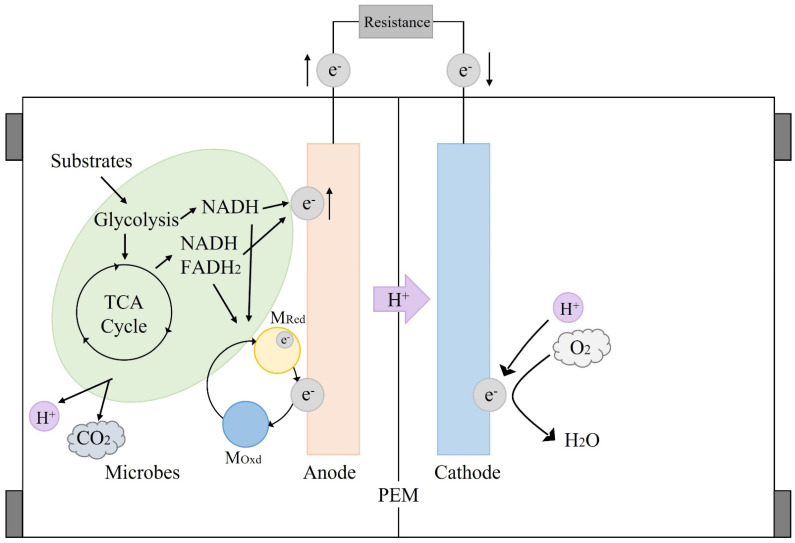
The operation mechanism of a dual-chamber MFC.

In recent years, the microbial fuel cell (MFC) technology has become one of the most representative research hotspots in the bioenergy field. It has been considered a promising solution with the sustainable potential to meet energy demands [3]. The MFC system works depending on the conversion of chemical energy to electrical energy supported by the metabolic activity of certain microbes (Figure 1). As a typical bioelectrochemical system, the MFC consists of an anode region and a cathode region separated by a proton exchange membrane (PEM). The electricity generation of MFCs relies on biological oxidation and oxygen reduction occurring in the anode and cathode regions, respectively. In the anode region, microbes act as biocatalysts to decompose substrates for the generation of electrons and protons through cellular respiration [4]. These electrons transported through the external circuit and protons transported through the PEM result in a reduction reaction with oxygen to generate water in the cathode region [5]. This energy generation process has many advantages, such as mild production conditions, simple operations, and a wide range of biocatalyst sources [6,7].

The application of microbial fuel cells is mainly combined with wastewater treatment [8]. It provides a feasible way to solve the issues of both water pollution and energy shortage. The discharge and accumulation of organic substances in wastewater can result in heavy pollution of water. At present, the widely used aerobic digestion treatment can efficiently decompose organic pollutants in wastewater into carbon dioxide under the action of microorganisms [9]. However, like other conventional wastewater treatment methods, this treatment still results in a lack of potential utilization of chemical energy in organic pollutants. These organic substances in wastewater have been considered available substrates for many strains [10]. As the microbes can use these organic pollutants to support metabolic activities and generate electrons, the MFC system can achieve simultaneous organic pollutant degradation and electricity production [11,12]. In addition, the MFC-based anaerobic digestion technology also avoids the higher energy consumption in conventional aerobic wastewater treatment methods [1,11].

**Figure 2:**
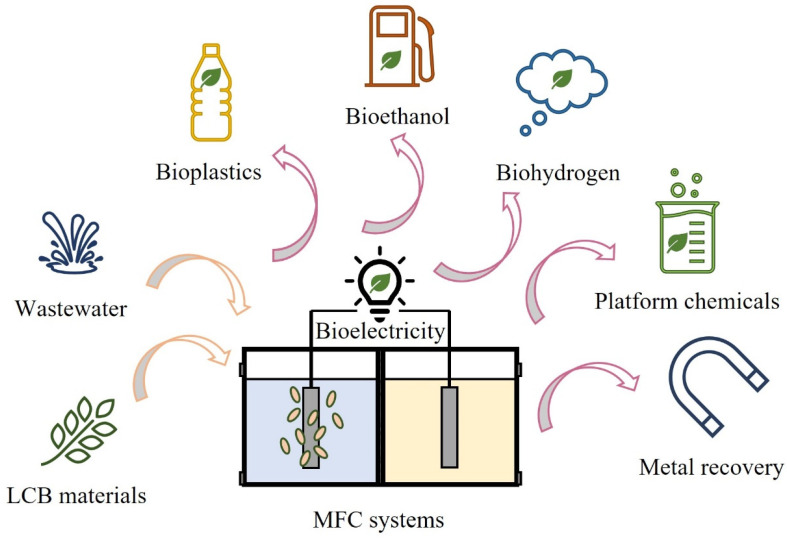
Waste valorization by MFC systems.

Currently, the application of microbial fuel cells also focuses on the simultaneous production of electricity and value-added products due to the diversity of strains and metabolic pathways [6,13]. Microbes can produce a variety of biofuels, volatile fatty acids, biopolymers, and other platform compounds through the fermentation process during the electricity generation of MFCs [14,15,16,17]. Furthermore, substrates for MFCs also extend from pure chemicals and organic wastewater to lignocellulosic biomass (LCB) due to the wide substrate availability of strains [18]. As one of the most abundant renewable resources, the annual production of LCB reaches about 200 billion tons [19]. LCB resources mainly exist in the form of agricultural and forestry wastes. Disposal and burning of such resources will cause a serious waste of resources and environmental pollution. However, the sugars generated by the hydrolysis of LCB are ideal carbon sources for the growth and metabolism of microbes. Similar to that of organic wastewater, the utilization of LCB hydrolysates as the substrate in MFCs can effectively achieve the recycling of biomass energy and the treatment of agricultural and forestry wastes. Therefore, the MFC system is a promising sustainable technology for simultaneous energy production and waste valorization (Figure 2).

The electrogenic capacity of MFCs generally depends on strains, substrates, electrode properties, and operating conditions. The application of electron mediators and the control of operating conditions can also improve the performance of the MFCs. Therefore, this review provides a detailed discussion of recent progress in these research fields. In addition, this review summarizes the recent advances of MFC systems in wastewater treatment, production of value-added products, and applications as biosensors. It also includes prospects for the future development of the MFC system.

## 2. The Selection and Modification of Strains

### 2.1. The Form of Cell Cultures

Substrate oxidation by microbes in the anode is the only source of electron generation in MFC systems. *Geobacter* and *Shewanella* are electrogenic microbes commonly used in MFCs [20]. Several yeast strains, such as *Saccharomyces cerevisiae*, *Candida melibiosica*, and *Kluyveromyces marxianus*, have also participated in the operation of MFC systems [21]. In addition, archaebacteria, cyanobacteria, and proteobacteria are promising strains for electricity generation [22]. However, the eukaryotic algae can act as both electron producers and acceptors in the anode and cathode, respectively [22]. The anodic inoculations of MFCs include pure cultures and mixed cultures. Pure cultures might achieve more efficient conversion of substrates to electricity because of simple and well-defined metabolic pathways. However, it also has higher requirements on the purity and concentration of the substrate. Therefore, selectivity for specific substrates might limit the ability of pure cultures to generate electricity using complex substrates such as wastewater and LCB hydrolysates. Currently, pure cultures mainly participate in studies on electricity generation performance and electron transport mechanisms of specific strains. However, Pandit et al. [23] developed a pure culture-based bioaugmentation strategy to improve the volumetric current density and shorten the start-up time of MFCs. Mixed cultures are advantageous for the scale-up of MFC systems due to their higher adaptability to complex substrates. The synergistic effect of various strains in the mixed culture might also be conducive to the efficient operation of MFC systems. Activated sludge is the most representative mixed culture used for MFC systems. The sludge pretreated with acid and heat can further enhance electricity generation [24]. However, the microbe composition of activated sludge is very complex. It is difficult to determine the precise pathway of substrate conversion. Therefore, the co-culture of defined strains might enhance the performance of MFC systems through synergy based on their specific functions. Schmitz and Rosenbaum [25] developed a co-culture scheme of *Pseudomonas aeruginosa* and *Enterobacter aerogenes*. The electron mediator produced by *Pseudomonas aeruginosa* can improve the electron transfer efficiency to the anode. This co-culture system can achieve an over 400% increase in electrical current generation under an optimized oxygen supply.

### 2.2. Strain Modification Based on Genetic Engineering

Although various wild-type strains have successfully achieved electricity production, it is still necessary to further improve the electrochemical activity of strains through suitable methods. Several physical and chemical methods have been considered as potential ways to enhance the ability of strains to generate electricity [26]. Genetic engineering is also a promising strategy to improve the electrochemical activity of strains. It mainly involves gene modification related to metabolic activity, the electro-shuttle pathway, and substrate utilization. The enhancement of extracellular electron transfer (EET) based on genetic engineering is an effective method to improve the performance of MFC systems. The modification of cytochrome c maturation can achieve a 77% increase in the current generation [27]. A constructed hybrid system of cytochrome c maturation can also increase the overall current by 121% [28]. In addition, the cytochrome OmcZs expressed by Escherichia coli can enhance the current production by binding riboflavin. Synthetic biology has played a role in enhancing the EET efficiency of strains. Liu et al. [29] enhanced the electricity output of the *Pseudomonas aerugino* strain based MFC by assembling type IV pili with high conductivity. Lin et al. [30] promoted the EET efficiency of *Shewanella oneidensis* by enhancing the biosynthesis and transportation of flavins. Kasai et al. [31] and Cheng et al. [32] also enhanced the EET of *Shewanella oneidensis* by improving the intracellular level of 3′,5′-cyclic adenosine monophosphate. Min et al. [33] developed an engineered *Shewanella oneidensis* strain with a gene cluster of flavin biosynthesis. This strain can achieve a 110% increase in the maximum current density of MFC systems. In addition, the increase in intracellular NAD(H/+) based on a modular synthetic biology strategy can improve both intracellular electron flux and EET efficiency [34]. McAnulty et al. [35] focused on the substrate expansion for power generation of MFC systems. They achieved the conversion of methane to electricity by developing a synthetic consortium with the main strains comprised of engineered *Methanosarcina acetivorans*, *Paracoccus denitrificans*, and *Geobacter sulfurreducens*. Li et al. [36] developed an engineered *Shewanella oneidensis* strain for electricity generation directly from xylose. It can achieve a maximum power density of 2.1 mW/m^2^. Genetic-engineering-based strain modification for enhancing electricity generation has achieved desired results in laboratory-scale MFC systems. However, there is still a lack of specific progress in the scale-up of MFC systems.

## 3. The Selection of Substrates for MFC Systems

### 3.1. Defined Substrates

Currently, defined substrates for MFC systems mainly include sugars and organic acids. Glucose is a common substrate for MFC systems. Christwardana et al. [37] used glucose for a yeast-based MFC to achieve a maximum power density of 374.4 mW/m^2^. However, Obileke et al. [4] pointed out that glucose might lead to low coulombic efficiency of MFC systems due to the electron loss caused by competition strains and the substrate consumption for fermentation. There are also studies using xylose as the substrate for MFC systems. Haavisto et al. [38] observed the highest power density of 333 mW/m^2^ using xylose for an up-flow MFC system. Li et al. [39] developed a microbial consortium consisting of engineered *Klebsiella pneumoniae* and *Shewanella oneidensis*. It can achieve a maximum power density of 104.7 mW/m^2^ using co-substrates of xylose and glucose. Several studies have compared the performance of MFC systems using different substrates. Ullah and Zeshan [40] studied the electricity generation of the double-chamber MFC using glucose, acetate, and sucrose, respectively. They reported a maximum power density of 91 mW/m^2^ using acetate as the most effective substrate. Jin et al. [41] also obtained similar results. They observed the best electricity production performance of the dual-chamber MFC using sodium acetate compared with glucose and lactose. In addition, acetates also have an advantage in the electrochemical performance of MFC compared to lactate and octanoate [42]. It might be related to the lower ohmic loss of the biofilm when using acetate as the substrate for MFC systems [42]. In recent years, acetate has also participated in the scheme of co-substrates with pollutants to improve the performance of MFCs in electricity generation and toxicant degradation. Shen et al. [43] obtained a voltage output of 389.0 mV with an initial phenol degradation of 78.8% using acetate as the co-substrate for the single-chamber MFC. Yu et al. [44] also used the dual-chamber MFC with acetate co-substrate to achieve increases of 4.3-fold in power generation and ∼42% in removal efficiency of 4-chlorophenol. In addition, Ndayisenga et al. [45] obtained increases of 60.1% in coulombic efficiency and 64.7% in microcystin-LR using acetate co-substrate for the dual-chamber MFC. Mancilio et al. [46] used acetate co-substrate to achieve a power density of 398 mW/m^2^ and a p-Coumaric acid degradation of 79%. However, they also reported higher potential and power density of the MFC using acetate as the single substrate.

### 3.2. Wastewater

Organic wastewater can provide essential nutrients for the growth and metabolism of microbes. This review focuses on the utilization of practical wastewater for MFC systems. There are studies using municipal wastewater as the substrate for MFCs. A natural microflora-based MFC system [47] achieved the maximum current density of 525 ± 20 mA/m^2^ and coulombic efficiency of 54% using 100% septic tank wastewater (STWW). Thulasinathan et al. [48] compared the electricity generation by *Cronobacter sakazakii* AATB3 and *Pseudomonas otitidis* AATB4 using STWW in the dual-chamber MFC. They observed a higher power density of 280 mW/m^2^ and a higher current density of 800 mA/m^2^ by *Pseudomonas otitidis* AATB4. This strain can also achieve the maximum coulombic efficiency of 15.5%. The study also compared the electricity generation by the co-culture of *Serratia marcescens* AATB1 and *Klebsiella pneumoniae* AATB2 and the single culture of each strain using STWW as the substrate [49]. It indicated that the co-culture has the best performance. It can achieve the maximum power density of 398.69 mW/m^2^ and the maximum current density of 869.11 mA/m^2^. Ramu et al. [50] focused on the food industry wastewater substrate for the MFC system. They used a *Klebsiella pneumoniae*-FA2 strain based MFC with food industry wastewater to obtain the maximum power generation of 428.71 mW/m^2^ and coulombic efficiency of 74.6%. Dairy wastewater is also an available substrate to achieve the maximum power density of 621.13 mW/m^2^ and the maximum current density of 795.74 mA/m^2^ [51]. In recent years, agro-processing wastewater for electricity generation has received extensive attention. Raychaudhuri and Behera [52] observed the maximum volumetric power density of 656.10 mW/m^3^ and coulombic efficiency of 17.21% using rice mill wastewater in the anode chamber with intermittent air exposure. Ng et al. [53] used the palm oil mill effluent (POME) to obtain the maximum power density of 0.45 mW/m^2^ by an algal-biophotovoltaic device. Islam et al. [54,55] studied electricity generation from POME by co-cultures. They observed the maximum power density of 12.87 W/m^3^ by the co-culture of *Klebsiella pneumonia* and *Lipomyces starkeyi* [54] and 14.78 W/m^3^ by the co-culture of *Pseudomonas aeruginosa* and *Klebsiella variicola* [55]. Sarmin et al. [56] also used POME to achieve a power density of 500 mW/m^2^ by the co-culture of *Saccharomyces cerevisiae*, *Klebsiella variicola*, and *Pseudomonas aeruginosa*. Zhang et al. [57] focused on electricity generation of the MFC system using molasses wastewater as the substrate. It can achieve a maximum power density of 1410.2 mW/m^2^. Naina et al. [58] observed the highest power density of 194.7 mW/m^2^ using distillery wastewater under the borate buffer environment. Several studies also focus on animal wastewater for electricity generation. Oyiwona et al. [59] obtained a volumetric power density of 6.9 W/m^3^ using poultry wastewater. Ren et al. [60] observed a power density of 33.3 mW/m^3^ using swine wastewater. Ni et al. [61] also used swine wastewater to achieve a power density of 770.97 mW/m^2^.

### 3.3. LCB Substrates

**Figure 3:**
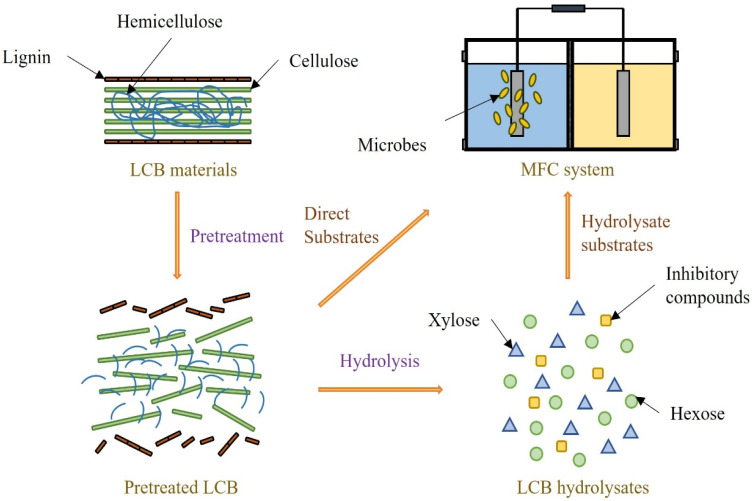
The utilization of LCB substrates for MFC systems.

As a renewable source rich in carbon, LCB exists mainly in the form of agricultural and forestry waste. LCB with appropriate pretreatment can participate in the operation of the MFC system as a hydrolysate substrate or direct substrate (Figure 3). The proper hydrolysis method can efficiently decompose the cellulose and hemicellulose contained in LCB into monosaccharides. These LCB hydrolysates containing a variety of hexoses and pentose have been considered promising substrates for cell growth and metabolism. Catal et al. [62] used the sulfuric acid hydrolysate of pinewood flour to achieve a voltage of 0.43 V at 1000 Ω external resistance of a single-chamber MFC. Jablonska et al. [63] obtained a power density of 54 mW/m^2^ using rapeseed straw hydrolysates produced by hydrothermal pretreatment and enzymatic hydrolysis. Gurav et al. [64] compared the electricity generation of a *Shewanella marisflavi* BBL25 strain based MFC using hydrolysates of barley straw, Miscanthus, and pine, respectively. As the most effective substrate, barley straw hydrolysate can achieve the maximum current output density of 6.850 mA/cm^2^ and the maximum power density of 52.80 mW/cm^2^. The authors also pointed out that barley straw hydrolysates lead to more elongated strain cells due to higher concentrations of lactate and formate. However, there are also studies directly using LCB materials as substrates for electricity generation. Simultaneous LCB degradation and electricity generation might rely on the combined action of multiple strains. Flimban et al. [65] studied the electricity generation of a dual-chamber MFC using the direct substrates of potato peels and rice straw, respectively. The power densities obtained from potato peels and rice straw can reach 152.55 mW/m^2^ and 119.35 mW/m^2^, respectively. Mohd Zaini Makhtar and Tajarudin [66] compared the electricity generation of a membrane-less MFC system using banana peel, corn bran, and POME. They observed a voltage generation of 237.1 mV with a power density of 23.75 mW/m^2^ achieved using the banana peel as the most effective substrate. Yoshimura et al. [67] developed a hydrodynamic cavitation system for the pretreatment of rice bran. They reported an increase of 26% in the total electricity generation using such pretreated rice bran because of the efficient substrate utilization. In addition, Jenol et al. [68] compared the electricity generation of a *Clostridium beijerinckii* SR1 strain based MFC using the direct substrate and hydrolysate substrate of sago hampas. The power density achieved from these two substrate forms of sago hampas can reach 73.8 mW/cm^2^ and 56.5 mW/cm^2^, respectively. Despite the potential of LCB for MFC-based biomass valorization, difficulties in collection and transportation limit the large-scale application of LCB.

## 4. The Electrode Modification for MFC Systems

### 4.1. Anode Modification

The modification of MFC anodes mainly focuses on improving the specific surface area and surface properties. Heat treatment and acid treatment are feasible surface treatment methods to increase the specific surface area of the anode [69]. Electrochemical oxidation methods can increase the specific surface area of the anode and introduce new functional groups to the anode surface [69,70]. These methods are all conducive to facilitating electrical contacts of strain cells to form electron-donating biofilms. However, more studies have used different materials for electrode modification to enhance the adhesion of strain cells and promote electron transfer to the anode surface. Metals and metal oxides have widely participated in anode modification. Xu et al. [71] studied the electricity generation of dual-chamber MFCs with carbon cloth anodes modified with MnO_2_, Pd, and Fe_3_O_4_, respectively. The maximum power densities achieved by anodes modified with such materials can reach 824, 782, and 728 mW/m^2^, respectively. The authors also pointed out that anodes modified with different materials lead to the enrichment of different strains on the anode surface. Yu et al. [72] observed the maximum power density of 29.98 mW/m^2^ using the anode modified with bentonite-Fe, and 18.28 mW/m^2^ using the anode modified with Fe_3_O_4_. They also reported increases in the stable voltage and decreases in the internal resistance of the MFCs with modified anodes compared to the bare graphite felt anode. The carbon cloth modified with cobalt oxide [73] and the nitrogen-doped carbon nanorods modified with Co-modified MoO_2_ nanoparticles [74] can also improve the electricity generation of MFCs. Li et al. [75] reported a positive effect of zero-valent iron on the improvement of maximum power density with structured biofilm and enriched functional microbial communities. However, they also observed the inhibition of electricity generation by the high concentration of zero-valent iron. Several studies have modified anodes with carbon materials, such as graphene oxide (GO) and carbon nanotubes (CNT). Paul et al. [76] used the carbon felt (CF) anode modified with GO and zeolite to achieve a 3.6-times higher power density and 2.75-times higher coulombic efficiency than having used the bare CF anode. They indicated the higher biocompatibility that originated from the improved specific surface area by graphene oxide and enhanced microbe adhesion by zeolite. The power densities achieved by the CF anode modified with GO and Fe_2_O_3_ are 1.72 times and 2.59 times that of MFCs with the graphene anode and the unmodified anode, respectively [77]. Liang et al. [78] pointed out that anodes modified with graphene, GO, and CNT have higher electrochemically active surface areas and enriched microbial communities. Zhang et al. [79] indicated that the graphite felt modified with CNT can promote biofilm growth and enhance electron transfer. In addition, polymers usually participate in anode modification combined with metal-type or carbon-type materials as composites. The power density achieved by the anode modified with polydopamine and reduced GO reaches 2.2 and 1.9 times that of MFCs with anodes modified with polydopamine and reduced GO, respectively [80]. The anode modified with polyaniline (PANI) and Au can also improve bioelectrochemical activity [81]. Mashkour et al. [82] pointed out the positive effect of PANI on biofilm growth. The CF anode modified with nitrogen-doped CNT, PANI, and MnO_2_ can achieve a 2.76-times higher cell biomass content than that of the bare anode [83].

### 4.2. Cathode Catalyst

The efficiency of cathode-based oxygen reduction directly affects the electricity generation of the MFC system. Appropriate cathode catalysts can improve the power output efficiency of MFC systems by promoting electron transfer and enhancing oxygen reduction. Platinum-based cathode catalysts can improve the activity of oxygen reduction [84]. However, they have limited availability in large-scale applications due to high costs and low stability [85]. Currently, more studies are attempting to develop nanocomposite-based cathode catalysts to enhance the electrochemical activity of MFC systems. Liu et al. [86] focused on the cathode catalysts based on metals and metal oxides. They developed an activated carbon cathode modified with Cu_2_O and Cu to achieve a peak power density of 16.12 W/m^2^. They also pointed out that the catalytic activity of Cu_2_O to oxygen reduction and the high electrical conductivity of Cu improve the performance of the cathode. Majidi et al. [87] observed a power density of 180 mW/m^2^ using a carbon cloth cathode modified with α-MnO_2_ nanowires and carbon Vulcan. Chiodoni et al. [88] also reported the positive effect of manganese-oxide-based cathode catalysts on MFC performance. Rout et al. [89] focused on the cathode catalysts combined with metal oxides and non-metal materials. They developed a nanocomposite of MnO_2_ and reduced GO to achieve a 2.7-times increase in volumetric power density. They also pointed out that this nanocomposite can provide a four-electron oxygen reduction pathway and enhance electron transfer. Mecheri et al. [90] reported that the cathode catalyst based on FePc and GO can improve the electrochemical performance of the MFC. The 3D composite of CNT and MoS_2_ has also been considered an available cathode catalyst for efficient oxygen reduction [91]. Li et al. [92] observed a maximum power density of 1177.31 mW/m^2^ and a current density of 6.73 A/m^2^ using the cathode catalyst of bacterial cellulose doped with P and Cu. They indicated that more active sites in this cathode catalyst improve the catalytic activity of oxygen reduction. Kaur et al. [93] developed a composite catalyst of PANI and an iron-based metal-organic framework. It can achieve a power density of 680 mW/m^2^ and a limiting current density of 3500 mA/m^2^. The Ni-based metal-organic framework has also been considered an efficient cathode catalyst to promote oxygen reduction [94]. In addition, the layered double hydroxide (LDH) has also participated in cathode catalyst development. Jiang et al. [95] developed a composite of Fe_3_O_4_ and NiFe-based LDH to achieve the maximum power density of 211.40 mW/m^2^. They indicated the advantages of LDH in terms of electroactive site availability, rate capability, and cycling stability. In another study, the composite catalyst of NiFe-based LDH and Co_3_O_4_ achieved the maximum power density of 467.35 mW/m^2^ [96]. Tajdid et al. [97] synthesized CoNiAl-based LDH. This material improved the performance of graphite cathode by working independently or combined with NiCo_2_O_4_. With the development of material science, co-composites based on various materials have become the choice for cathode catalyst development [98,99]. These co-composite cathode catalysts can exploit the specific advantages of each material. It is conducive to improving the comprehensive electrochemical performance of MFC systems, including electron transfer efficiency, oxygen reduction catalytic efficiency, and operation stability.

## 5. The Operation Environment of MFC Systems

### 5.1. Electron Transfer Mediators

EET in MFC systems includes direct electron transfer and mediated electron transfer. Several strains, such as *Geobacter* and *Shewanella*, can transfer electrons directly to the anode surface via intricate networks of outer membrane cytochromes [100,101]. However, more strains need redox mediators for electron transfer due to the lack of electrochemically active surface proteins [102]. Electron transfer mediators acquire electrons within strain cells and transfer the electrons to the cathode surface. They can achieve continuous electron transfer via the conversion of oxidized and reduced states. A variety of organic compounds are common artificial exogenous mediators for electron transfer in MFC systems. Pal and Sharma [103] studied the electricity generation of a *Pichia fermentans* strain based MFC with the mediator of methylene blue (MB). They reported higher maximum power densities of both single- and dual-chamber MFCs containing MB than those without mediators. MB can also achieve a 1.22-fold increase in the steady-state voltage of a dual-chamber MFC [104]. Christwardana et al. [105] compared the electricity generation of a *Saccharomyces cerevisiae* strain based MFC with the addition of MB and methyl red. They pointed out a more efficient electron transfer of MB due to more effective capture by yeast and higher electron collection. MB also has advantages in enhancing the electricity generation of MFC systems compared to congo red and crystal violet [106]. Chauhan et al. [107] reported positive effects of both MB and methyl orange on the electricity generation of a dual-chamber MFC. Chen et al. [108] reported a ~400% increase in coulombic efficiency of a dual-chamber MFC using neutral red (NR) as the mediator. They pointed out that the proper concentration of NR can improve electricity transfer efficiency and promote the growth of the exoelectrogens. Moreno et al. [109] also observed the positive effect of NR on the electricity generation of continuous flow MFCs. The addition of NR can improve the maximum power density from 777.8 mW/m^3^ to 1428.6 mW/m^3^ and the maximum current density from 3444.4 mA/m^3^ to 5714.3 mA/m^3^. Marcílio et al. [110] used methylene green as the mediator to achieve a 20% increase in the voltage of an acetate-fed MFC with a stable operation for about six days. In addition, metabolites of specific microbes can also act as endogenous mediators to participate in the electron transfer [20]. Ajunwa et al. [111] determined the electricity generation of the glucose-fed MFC with flavins and pyocyanin as mediators. These endogenous mediators can improve the power production efficiency of MFC systems by simplifying the electron transfer process.

### 5.2. Operation Conditions of MFC Systems

As with microbial fermentation, microbial activity in MFC depends on multiple operating parameters. Mechanistic studies and parameter optimization of various operating conditions are conducive to further improving the performance of MFCs. The temperature is considered one of the primary conditions affecting the electrical power generation of MFC systems due to its significant influences on the metabolic activity of microbes, mass transfer efficiency, and thermodynamic properties. The increased temperature can increase power density and reduce internal resistance due to an improved conductivity [112,113,114]. However, higher temperatures also negatively affect microbial activity, membrane stability, and partial pressure of oxygen [4]. The temperature range of 30 to 45 °C is conducive to maintaining growth efficiency and electrochemical activity of microbes in the MFC system [114]. Environment or room temperature is generally the operating temperature of MFCs, although it might reduce the efficiency of electrical power generation. However, Heidrich et al. [115] reported a minor effect of low temperature on the power density of MFCs due to the potential self-heating performance of MFC biofilms. Gonzalez-Martínez et al. [116] studied the performance of the MFC system at 25 °C and 8 °C, respectively. They observed a difference in bacterial communities but similar voltage at these temperatures. It indicates the potential for stable MFC operation at lower temperatures. The pH is the other primary condition affecting the performance of MFC systems. The promotion of proton transfer from anode to cathode in MFC systems usually depends on the different pH of the anode and cathode. However, the limited proton transfer efficiency of the PEM might lead to a decrease in anodic pH due to proton accumulation and an increase in cathodic pH due to a lack of protons. The lower anolyte pH might result in lower electron generation efficiency due to the inhibition of the growth and metabolism of microbes. The higher catholyte pH might decrease oxygen reduction efficiency [117]. Therefore, the unstable anodic and cathodic pH might reduce the power production efficiency of MFC systems. Phosphate- [118] and borate-based [58] buffers can effectively maintain the electrolyte pH of MFCs. HCO_3_^−^/H_2_CO_3_ buffer systems based on anolyte or catholyte recirculation are promising alternatives to phosphate-based buffers [119,120]. There are also studies focusing on effects of initial substrate concentration, aeration rate, and hydraulic retention time [121,122]. Promoting cell growth and metabolism is still the primary solution to enhance MFC performance by controlling MFC operating conditions. In addition, the parameter optimization for the operating conditions of MFC systems can further improve power generation efficiency (Table 1).

## 6. Recent Progress in the Application of MFC Technology

### 6.1. Wastewater Treatment

Redox reactions based on MFC systems have achieved simultaneous chemical oxygen demand (COD) removal and electricity generation from wastewater. COD removal efficiency and maximum power density are the main parameters to describe the performance of MFC systems in wastewater treatment. Currently, both single- and dual-chamber MFC systems perform good COD removal efficiencies. Dual-chamber MFCs can achieve COD removal efficiencies of 79.8% [129], 83% [130], and 94.6% [131] from sugar wastewater, seafood processing wastewater, and brewery wastewater, respectively. Similarly, single-chamber MFCs can achieve COD removal efficiencies of 88% [132], 90% [133], and 96% [134] from tannery wastewater, wastewater of fish markets, and dairy wastewater, respectively. Constructed wetland (CW) MFC systems have also focused on wastewater treatment. The COD removal efficiency can reach 70% from dyestuff wastewater using the CW dual-chamber MFC [135] and 79.83% from Zn (II) contaminated wastewater using the CW single-chamber MFC [136]. However, there are significant differences in the maximum power density of these MFC systems. Zhang and Liu [137] reported the positive effect of electrode modification on the performance of MFCs in wastewater treatment. They obtained a COD removal capacity of 3.07 kg COD/m^3^/d and a maximum power density of >1680 mW/m^3^ from coking wastewater using a dual-chamber MFC–membrane bioreactor system with a modified granular activated carbon cathode and catalytic cathode membrane. Kadivarian et al. [138] studied the COD removal and electricity generation of single-chamber MFC packs with parallel and serial connections. They pointed out that the serial connection of MFCs can achieve a higher COD removal efficiency while the parallel connection of MFCs can achieve a higher power density. Therefore, the performance of MFC systems in COD removal and electricity generation depends on the combined effect of multiple factors. In recent years, wastewater treatment based on MFC systems has also focused on certain pollutants. Xia et al. [139] used the dual-chamber MFC to achieve a power density of 543.75 mW/m^2^ with 76.15% total nitrogen removal and 83.23% ammonia-nitrogen removal from organic acid fermentation wastewater. Zeng et al. [140] also achieved a nitrogen removal of 63.4% using a three-phase single-chamber MFC with a phase of immobilized Halomonas strain. However, Srikanth et al. [141] obtained a power density of 225 mW/m^2^ with removal efficiencies of 95% oil, 80% phenol, and 79.5% sulfide using the single-chamber MFC in a continuous mode. The capacity of MFC systems to remove different pollutants depends on the metabolic pathway and activity of strains. In addition, there are also studies using the MFC system to achieve simultaneous wastewater treatment and the recovery of heavy metals, such as silver [142] and copper [143].

### 6.2. The Production of Value-Added Products

The production of value-added products from various organic wastes has achieved significant progress using conventional fermentation equipment. However, the application of MFC technology in this field is still limited. In the anaerobic environment of the anode chamber, microbes cannot fully oxidize substrates to CO_2_ to generate electrons. Substrates might also flow into the fermentation pathway to generate anaerobic metabolites. Therefore, the MFC system can achieve integrated production of electricity and value-added products by combining fermentation and electrochemical processes. As typical bioenergy, bioethanol originates from the conversion of sugars by ethanologenic strains in anaerobic fermentation. The operation of yeast-based MFCs can achieve simultaneous production of bioethanol. Birjandi et al. [144] used a *Saccharomyces cerevisiae* strain based MFC to obtain a maximum ethanol production of 11.52 g/L with a maximum power density of 30.46 mW/m^2^ from glucose. There are a variety of wild-type and engineered strains that can utilize different sugars for ethanol production. Metabolic engineering and mixed culture technologies can achieve an integrated MFC-based system for ethanol production and electricity generation from LCB hydrolysates and sugar-containing wastewaters with complex sugar compositions and concentrations. In addition, the MFC system can achieve efficient conversion of LCB substrates to ethanol and electricity with the combination of advanced fermentation strategies, such as simultaneous saccharification and fermentation (SSF) and simultaneous saccharification and co-fermentation (SSCF). Moradian et al. [145] focus on the integrated production of electricity and gaseous bioenergy using MFC systems. They isolated the yeast *Cystobasidium slooffiae* strain JSUX1 from the activated sludge. This strain can achieve a power output of 67 mW/m^2^ and a hydrogen production of 23 L/m^3^ from xylose in the anode chamber of a dual-chamber MFC. With the increased focus on bioplastics, there are also attempts to produce polyhydroxybutyrate (PHB) using MFC systems. Lee et al. [17] developed an engineered *Shewanella marisflavi* BBL25 strain inserting polyhydroxyalkanoate synthesis genes from *Ralstonia eutropha* H16. This strain can achieve a PHB production of 6.31 g/L with a maximum current output density of 1.71 mA/cm^2^ from barley straw hydrolysates in the anode chamber of a dual-chamber MFC. However, Srikanth et al. [146] achieved a PHB production of 19% dry cell weight from synthetic wastewater in the cathode chamber. They indicated that the low level of dissolved oxygen resulting from the oxygen reduction in the cathode chamber is conducive to PHB accumulation. For the metabolic process in the anode chamber, the generation of metabolites and electricity might be affected by organic loading. Kondaveeti et al. [16] studied the production of electricity and volatile fatty acids (VFAs) from citrus peel extract using a single-chamber MFC. They reported that a high level of electricity generation can be achieved at low organic loading, while a high production of VFAs can be achieved at high organic loading.

### 6.3. The Application of MFC-Based Biosensors

The MFC has been considered a promising system for biosensors. According to the current flow of MFC affected by biological activities, MFC-based biosensors can reflect various conditions of liquid samples, such as biochemical oxygen demand (BOD) and toxicity [117]. The operation of MFC-based BOD biosensors depends on the positive linear correlation between the electrical current output and the BOD value in a specific range. Karube et al. [35] were the first to confirm the availability of biosensors based on the MFC system to determine the BOD concentration of the wastewater. In recent years, there have been several MFC-based biosensors for BOD measurement. Commault et al. [147] developed the MFC-based biosensor with *Geobacter*-dominated biofilms to determine the BOD of milk over 17.5 h. This biosensor can also achieve reproducibility of 94% with only a 7.4% error during milk BOD determination, compared with the conventional BOD_5_ method. It indicates the potential of MFC-based biosensors to accurately determine the BOD of dairy wastewater in a much shorter period. Hsieh and Chung [148] developed the MFC-based biosensor with a mixed culture of six strains to determine BOD concentrations lower than 240 mg/L in practical wastewater. They also indicated high reproducibility and stability of this biosensor in long operation periods. Similarly, a self-powered floating MFC-based biosensor based on MFC systems can achieve an autonomous operation for 150 days for monitoring and early warning of water pollutants [149]. In addition, the development of MFC-based BOD biosensors has also focused on the selectivity of specific compounds [150]. The toxicity detection of MFC-based biosensors usually depends on the inhibitory effect of toxic substances on cell metabolism. Therefore, the electrical current output usually has a negative linear correlation with the concentration of cytotoxic substances in a specific range. Some studies have developed MFC-based biosensors based on this mechanism to detect antibiotics [151] and organic toxicants [152]. Yu et al. [153] also used the MFC-based biosensor to determine the biotoxicity response of Cu^2+^, Hg^2+^, Zn^2+^, Cd^2+^, Pb^2+^, and Cr^3+^. In addition, this mechanism and function might also extend to the determination of pH values [154]. However, the toxicity detection of MFC-based biosensors might also involve other actions of microbes. The detection of Cr^6+^ depends on the electron competition between the anode and the Cr^6+^ reduction by Cr^6+^-reducing anaerobes, such as *Ochrobactrum anthropi* YC152 [155] and *Exiguobacterium aestuarii* YC211 [156]. Furthermore, there are also MFC-based biosensors based on the positive linear correlation between the electrical current output and some substances, such as Fe^2+^ [157] and p-nitrophenol [158]. Guo et al. [159] focused on maintaining MFC-based biosensors for long-term use. They reported that implementing hibernations of electroactive bacteria is an available maintenance method to efficiently recover the accurate BOD detection of MFC-based biosensors after their lay-up periods.

## 7. Future Perspectives

The MFC technology has shown potential as an integrated system for sustainable energy recycling, waste treatment, and biomass valorization. Lab-scale MFC systems have met the primary requirement for enhanced electricity generation. They have also achieved considerable progress in COD removal, the production of value-added products, and biosensor applications. However, there are still many challenges in the scale-up and commercialization of MFC systems.

The low output power of the MFC system is still one of the main problems in this field. The electricity generation of MFC systems is directly affected by the metabolic activity of microbes. Therefore, the screening of high-performance electrogenic strains and the strain modification based on genetic manipulation are still effective methods to improve the performance of electrogenicity by microbes. With the further development of genetic engineering and synthetic biology technology, the gradual replacement of wild-type strains with engineered strains might be the future trend of strain selection for MFC systems. In addition, the development of electrode materials and the modification of electrode structure are also effective methods to enhance the electricity generation of MFC systems. The main aim of electrode improvement is to increase microbial adhesion and electron transfer efficiency. Therefore, the developments of electrodes for MFCs still need to focus on promotions in electrical conductivity, surface area, and microbial affinity. Currently, the high cost of electrode materials is also one of the main reasons limiting the commercialization of MFC systems. The production of high-performance electrode materials from wastes and renewable natural feedstocks will be a way to solve this problem in the future. In addition, 3D technology has great application potential in improving the electrode surface structure. However, electrode modification also needs to focus on other physical and chemical properties to maintain stability in various substrate environments while improving electrical performance.

The operation of MFC systems relies on the utilization of substrates by microbes. Therefore, the selection of substrates needs to focus on the utilization efficiency of specific substrates by specific strains. Currently, more studies have determined the capabilities of wastewater treatment and power generation by MFC systems using synthetic wastewater. However, it is still necessary to further study the adaptability of different strains to the complex environment of composition and concentrations in practical wastewater. The recalcitrance of lignin is a challenge for the large-scale bioconversion of LCB. Efficient pretreatment methods still need to be developed to improve the substrate availability of LCB. For LCB hydrolysates, sufficient removal of cytotoxic substances might be conducive to improving the metabolic activity of strains but leads to an increase in the process costs. Although strains with tolerance to such cytotoxic substances might be a way to reduce such costs, the performance of these strains in MFC systems still needs to be determined. In addition, the selection of substrates and strains needs to aim at the specific function and application of MFC systems. For MFC systems focusing on electricity generation, more substrates need to be utilized for oxidation and electron generation instead of the conversion to unnecessary metabolites through other metabolic pathways. In contrast, if the priority of the MFC system is to produce value-added products, more substrates need to be converted to desired products rather than oxidation. The proper metabolic regulation might be an effective way to enhance the performance of strains consuming specific substrates for specific functions and applications of MFC systems. The better performance of MFC systems relies on the better fitness between strains, substrates, and functions.

The efficient operation of the MFC system depends on a stable internal and external environment. The scale-up of MFC systems needs to be supported by proper control of operating conditions. The temperature is still the main external factor affecting the performance of MFC systems. The low-temperature environment might lead to longer start-up times and lower operating efficiency of MFCs. Therefore, technical improvement of MFC systems is still needed to achieve stable performance in a wide range of temperatures. As for internal factors, such as pH, cell biomass concentration, substrate concentration, and electron mediator concentration, parametric optimizations based on experimental design and mathematic modeling are helpful. For the maintenance of MFC devices, growth inhibition and periodic removal of non-electroactive biofilms on the cathode are conducive to maintaining oxygen reduction efficiency during long-term MFC operation. However, the feasibility of this approach in the large-scale application of MFCs has not been clear. In addition, there are technical difficulties in maintaining the activity of anodic electroactive microbes for a long time in the scale-up of MFC systems. Therefore, it is necessary to develop convenient maintenance methods for MFC devices. The operation and maintenance of MFCs need to focus on maintaining high energy production efficiency and reducing energy loss.

## 8. Conclusions

As an emerging energy technology, the MFC system realizes the effective combination of electricity production, waste treatment, and biomass valorization. Mixed culture techniques and genetic engineering strategies are conducive to improving the substrate availability and electricity generation of electrochemically active microbes. Wastewater and LCB materials are promising substrates to achieve efficient operation of MFC systems and reduce substrate costs. Anode and cathode modifications based on different materials can improve the electricity output of MFC systems by enhancing extracellular electron transfer and oxygen reduction efficiency, respectively. The participation of the electron transfer mediators and the optimization of operating conditions have also improved the performance of MFC systems. Despite the challenges, MFC systems still have significant potential for scale-up for various applications.

## Figures and Tables

**Table 1:** Parametric optimization for enhancing MFC performance.

MFC Type	Microbial Community	Experimental Design	Optimized Conditions	MFC Performance	Reference
Dual-chamber MFC	Co-culture of *Klebsiella variicola* and *Pseudomonas aeruginosa*	Box–Behnken design	Inoculum composition: 1:1, Initial COD: 26.690 mg/L, pH: 7.21, Time: 15.50 days.	Power density: 12.21 mW/m^3^	[123]
Dual-chamber MFC	Anaerobic sludge	Central composite design	Degree of sulfonation: 68%, Aeration: 121.62 mL/min, Pt load: 0.42 mg/cm^2^.	Power density: 58.19 mW/m^2^	[124]
Cubical ceramic-based MFC	Sludge and human urine in a 1:1 ratio	Box–Behnken design	Membrane thickness: 1.55 mm, External resistance: 895.59 Ω, Anode area: 165.72 cm^2^.	Maximum absolute power generation: 467.63 μW	[125]
Dual-chamber MFC	*Acinetobacter pitii*	Central composite design	Initial dye: 295 ppm, pH: 7.5, Time: 71.27 h	Current density: 1.06 A/m^3^	[126]
Microalgae MFC	Immobilized *Saccharomyces cerevisiae* yeast	Central composite design	Yeast: content: 10.89% *w*/*v* Wastewater concentration: 56.94%	Power density: 11.25 mW/m^2^	[127]
Fabricated tubular MFC	Microorganisms in wastewater	Box–Behnken design	pH: 7, Substrate concentration: 75% Anode material: graphite rod	Maximum power density: 126 mW/m^2^	[128]

## Data Availability

Not applicable.

## Abbreviations

BOD	biochemical oxygen demand
CF	carbon felt
CNT	carbon nanotubes
COD	chemical oxygen demand
CW	constructed wetland
EET	extracellular electron transfer
GO	graphene oxide
LCB	lignocellulosic biomass
LDH	layered double hydroxide
MB	methylene blue
MFC	microbial fuel cell
NR	neutral red
PANI	polyaniline
PEM	proton exchange membrane
PHB	polyhydroxybutyrate
POME	palm oil mill effluent
SSCF	simultaneous saccharification and co-fermentation
SSF	simultaneous saccharification and fermentation
STWW	septic tank wastewater
VFAs	volatile fatty acids
